# A copula-based geo-additive approach to childhood health outcomes in five West African nations

**DOI:** 10.3389/fpubh.2026.1871312

**Published:** 2026-10-05

**Authors:** Reshav Beni, Shaun Ramroop, Faustin Habyarimana

**Affiliations:** School of Agriculture and Science, University of KwaZulu-Natal, Pietermaritzburg, South Africa

**Keywords:** anemia, composite index of anthropometric failure, geo-additive joint model, malnutrition, public health, West Africa

## Abstract

**Background:**

Anemia and malnutrition are major contributors to child morbidity and mortality in Sub-Saharan Africa, particularly in West Africa where their prevalence remains high due to multifaceted socio-economic and environmental factors. Malnutrition, as defined by WHO, includes both undernutrition and overnutrition and is linked to increased risk of non-communicable diseases. Anemia, characterized by low hemoglobin levels, impairs oxygen transport and is associated with developmental delays and increased mortality in children. Given their interrelated nature, a joint modeling approach is essential to understand shared risk factors and inform targeted interventions.

**Methods:**

This study analyses pooled DHS data from Gabon, Gambia, Liberia, Mauritania, and Nigeria to identify determinants of anemia and malnutrition. Malnutrition is measured using the Composite Index of Anthropometric Failure (CIAF), which integrates stunting, underweight, and wasting for a comprehensive assessment. A copula-based geo-additive joint model is employed to simultaneously examine both conditions, accounting for spatial variation and interdependence. This approach offers a novel framework for informing policy and improving child health outcomes in West Africa.

**Results:**

Key predictors of malnutrition and anemia were identified across five West African countries. Malnutrition risk was significantly associated with maternal education, water source, household wealth, child age, gender, and feeding practices. Notably, children from households using non-bottled water sources and those given milk were at higher risk. Several water-source categories were positively associated with malnutrition relative to bottled/sachet water; however, these associations may reflect socioeconomic and residential differences in the reference group and warrant cautious, non-causal interpretation. Anemia risk was significantly lower among urban children and those whose mothers had higher education. Fortified food and egg consumption were protective against anemia, while poverty and poor water sources increased risk. These findings highlight distinct and overlapping determinants of both conditions.

**Conclusion:**

This study highlights the complex interplay of socioeconomic, environmental, and dietary factors influencing malnutrition and anemia among children in West Africa. Key predictors include maternal education, household wealth, water source, and feeding practices, with notable geographic variation. Addressing these challenges requires integrated, multisectoral strategies focused on improving maternal education, access to safe water, dietary diversity, and poverty reduction. Geographically targeted interventions and strengthened health systems are essential.

## Introduction

In Sub-Saharan Africa, anemia represents a significant complication contributing to elevated rates of infant mortality and morbidity among children diagnosed with malnutrition (1, 2). The World Health Organization (WHO) defines malnutrition as “deficiencies or excesses in nutrient intake, imbalance of essential nutrients or impaired nutrient utilization” (3). Malnutrition encompasses undernutrition—including stunting, wasting, underweight, and micronutrient deficiencies—as well as overweight, obesity, and diet-related non-communicable diseases. According to the 2025 UNICEF/WHO/World Bank Joint Child Malnutrition Estimates, approximately 150.2 m children under 5 years of age were affected by stunting, 42.8 m by wasting, and 35.5 m by overweight in 2024. Despite progress over recent decades, the world remains off track to achieve the Sustainable Development Goal targets for ending all forms of malnutrition by 2030. Furthermore, nearly half of deaths among children under five are linked to undernutrition, with most occurring in low- and middle-income countries (38). This trendv is particularly pronounced in West Africa, where malnutrition prevalence remains high due to a complex interplay of individual, social, economic, political, and environmental factors (4).

Anemia, as defined by WHO, is a condition characterized by a reduction in the total number of red blood cells (RBCs) or hemoglobin levels in the blood, impairing the blood’s capacity to transport oxygen to tissues and organs (5). Anemia remains a substantial global public-health problem among young children. Children under 5 years of age are especially vulnerable to anemia because their rapid physical and cognitive development increases the demand for iron and other essential micronutrients. Inadequate dietary intake, poor nutrient absorption, and recurrent infections may further increase their risk of developing anemia (39).

The most recent WHO global estimate indicates that 39.8% of children aged 6–59 months were affected by anemia in 2019, equivalent to approximately two in every five children in this age group (40). In infancy and early childhood, anemia is associated with impaired cognitive development, growth retardation, weakened immune function, and increased mortality risk (1). Recognizing its severe health implications, WHO designated anemia as a global public health concern in 2004 (6).

Although separate marginal models can identify factors associated with anemia and malnutrition individually, they do not explicitly estimate the residual dependence between the two outcomes after adjustment for observed covariates. A copula-based joint regression model retains a separate marginal equation and link function for each outcome while linking the margins through a flexible dependence structure, thereby enabling simultaneous estimation of outcome-specific associations and the remaining dependence between the conditions. Where meaningful residual dependence is present, joint estimation may improve parameter precision or model fit by using information across the outcome equations; however, the magnitude of any efficiency gain is data- and model-dependent. Because a formal comparison with separately fitted marginal models was not undertaken in this study, the joint framework is motivated primarily by its ability to quantify the conditional dependence between anemia and malnutrition rather than by an assumed or quantified reduction in standard errors (41, 42).

Previous studies (4, 7–11) have predominantly relied on conventional anthropometric indices—such as stunting, underweight, and wasting—as proposed by WHO (12). While these indices provide valuable insights, they often fail to capture the full scope of childhood malnutrition when considered in isolation. Each index measures a distinct aspect of malnutrition, potentially overlooking children experiencing multiple forms of malnutrition simultaneously (13). To address this limitation, the Composite Index of Anthropometric Failure (CIAF) has been proposed as a more holistic measure. By integrating multiple anthropometric indicators, CIAF offers a more nuanced and accurate assessment of childhood malnutrition, reducing the risk of misclassification or underestimation of its true burden (13, 14).

This study investigates the determinants of childhood anemia and malnutrition, measured using the Composite Index of Anthropometric Failure, across Gabon, Gambia, Liberia, Mauritania, and Nigeria. Its principal methodological contribution is the application of a copula-based geo-additive model that analyses the two outcomes jointly rather than treating them as independent conditions. The framework accounts for residual dependence between anemia and malnutrition while accommodating linear, nonlinear, and geographical effects. In addition, CIAF captures both single and overlapping anthropometric failures, providing a more comprehensive assessment of childhood undernutrition than separate indicators of stunting, wasting, or underweight. Evidence from multi-country studies combining a comprehensive malnutrition measure with joint copula modeling and flexible geographical effects remains limited. The study consequently offers a more integrated understanding of the shared and outcome-specific factors associated with childhood anemia and malnutrition, with potential relevance for coordinated and geographically targeted policy interventions.

These countries were selected due to the scarcity of research on the topic and the availability of up-to-date data, enabling a timely and meaningful analysis. While previous studies (7, 8) primarily focused on sociodemographic factors, this study adopts a more comprehensive approach by incorporating dietary factors (e.g., consumption of milk, formula feeds, tubers, eggs, meat), sanitation variables (e.g., access to clean water and toilet facilities), clinical variables (e.g., fever, cough), and geographic coordinates. This multifaceted methodology enhances the understanding of the complex determinants of malnutrition and anemia, thereby improving intervention strategies in Sub-Saharan Africa.

## Data

The data for this secondary analysis were obtained from the Demographic and Health Surveys (DHS) conducted in selected West African countries. The DHS employs a complex sampling design that incorporates both stratification and clustering in two stages, with unequal probabilities of selection. This design results in a weighted sample, ensuring that the estimates remain nationally representative.

Following the guidelines outlined in the DHS manual, the data were extracted from the Kids Recode dataset, which focuses on children under the age of five born to women who participated in the survey. To facilitate a comprehensive analysis, a pooled dataset was constructed by integrating data from multiple survey waves conducted in Gabon (2019–2021), Gambia (2019–2020), Liberia (2019–2020), Mauritania (2019–2021), and Nigeria (2021).

The pooled dataset initially comprised 65,994 observations from five countries. Observations with missing data for variables required in the analysis were excluded using a complete-case approach. A total of 4,209 observations (6.4%) were removed because of missing information, resulting in a final analytical sample of 61,785 observations (93.6% of the initial sample).

The final sample comprised 12,975 observations from Gabon (21%), 12,357 from The Gambia (20%), 11,739 from Liberia (19%), 9,268 from Mauritania (15%), and 15,446 from Nigeria (25%). Nigeria contributed the largest proportion of observations, whereas Mauritania contributed the smallest. The country-specific sample sizes summed to the final analytical sample of 61,785 observations.

### Independent variables

This study examined a range of demographic, socioeconomic, and environmental factors that have been widely recognized in previous research as key determinants of child malnutrition and anemia. These factors guided the selection of independent variables, which included mother’s age, type of residence, maternal education level, source of drinking water, type of toilet facility, access to electricity, gender and age of the household head, type of cooking fuel, household wealth status, and use of mosquito bed nets. Additionally, child-specific variables such as dietary practices (e.g., consumption of milk, formula, fortified food, tubers, eggs, or meat), health facility visits, weight, age and recent illnesses (e.g., fever or cough) were incorporated into the analysis. An assessment of potential interaction effects within the copula-based geo-additive model revealed no statistically significant interactions.

The theoretical framework for this study is grounded in established literature, including works by Duru et al. (7), Igbokwe et al. (8), Oyekale (9), Sawadogo et al. (10), Adeyemi et al. (1), Gaston et al. (15) and Khulu et al. (2). This framework integrates well-established theories and empirical findings to guide the selection of explanatory variables hypothesized to influence malnutrition and anemia. It provides a structured approach to understanding the intricate relationships between demographic, socioeconomic, and environmental determinants of child malnutrition and anemia within the study region.

### Dependent variable

The Composite Index of Anthropometric Failure (CIAF), first introduced by (16), provides a comprehensive framework for assessing nutritional status by incorporating multiple dimensions of malnutrition into a single index. This approach overcomes a key limitation of traditional anthropometric indices—such as stunting, underweight, and wasting—that are typically analyzed in isolation, potentially underestimating the true extent of malnutrition by failing to account for overlapping forms of undernutrition (16). The conventional reliance on individual indices can obscure the overall prevalence of anthropometric failure within a population, as it does not reflect the simultaneous occurrence of multiple malnutrition conditions (17).

As outlined in the *Handbook of Anthropometry: Physical Measures of Human Form in Health and Disease* (18), the CIAF integrates three key anthropometric indicators—weight-for-age (WAZ), height-for-age (HAZ), and weight-for-height (WHZ)—to assess the nutritional status of children under 5 years old. This index classifies children into the following categories: (A) no failure (normal); (B) wasting only; (C) wasting and underweight; (D) wasting, underweight, and stunting; (E) underweight and stunting; (F) stunting only; and (Y) underweight only (14, 17).

By consolidating these categories, the CIAF offers a holistic perspective on anthropometric failure, capturing the full spectrum of undernutrition. This comprehensive approach provides a more accurate estimation of the malnutrition burden within a population, particularly by identifying children experiencing multiple nutritional deficiencies concurrently. The total anthropometric failure rate is determined by summing categories B, C, D, E, F, and Y, which collectively represent all cases of undernutrition (16, 17).Table 1 provides a detailed description of the various categories of CIAF [(14, 43)].

**Table 1:** Category Anthropometric Failure in under 5 year using CIAF.

Group	CIAF categories	Description	Wasting	Stunting	Underweight
A	No failure	Children whose height and weight are above the age-specific norm and who do not suffer from any anthropometric failure	No	No	No
B	Wasting only	Children with acceptable weight and height for their age, but who have subnormal weight for height	Yes	No	No
C	Wasting and underweight	Children with above-norm heights, but whose weight for age and weight for height are too low	Yes	No	Yes
D	Wasting, underweight and stunting	Children who suffer from anthropometric failure on all three measures	Yes	Yes	Yes
E	Stunting and underweight	Children with low weight for age and low height for age, but who have acceptable weight for their height	No	Yes	Yes
F	Stunting only	Children with low height for age but who have acceptable weight, both for their age and for their short height	No	Yes	No
Y	Underweight only	Children who are only underweight—whose weight for age is acceptable	No	No	Yes

In conclusion, the Composite Index of Anthropometric Failure (CIAF) serves as a comprehensive and robust tool for identifying various forms of anthropometric failure. By providing a more nuanced assessment of childhood malnutrition, the CIAF enhances our understanding of its multifaceted nature and supports the development of more targeted and effective intervention strategies (13).

Figures 1, 2 display the distribution of CIAF for the pooled and country level data sample.

**Figure 1:**
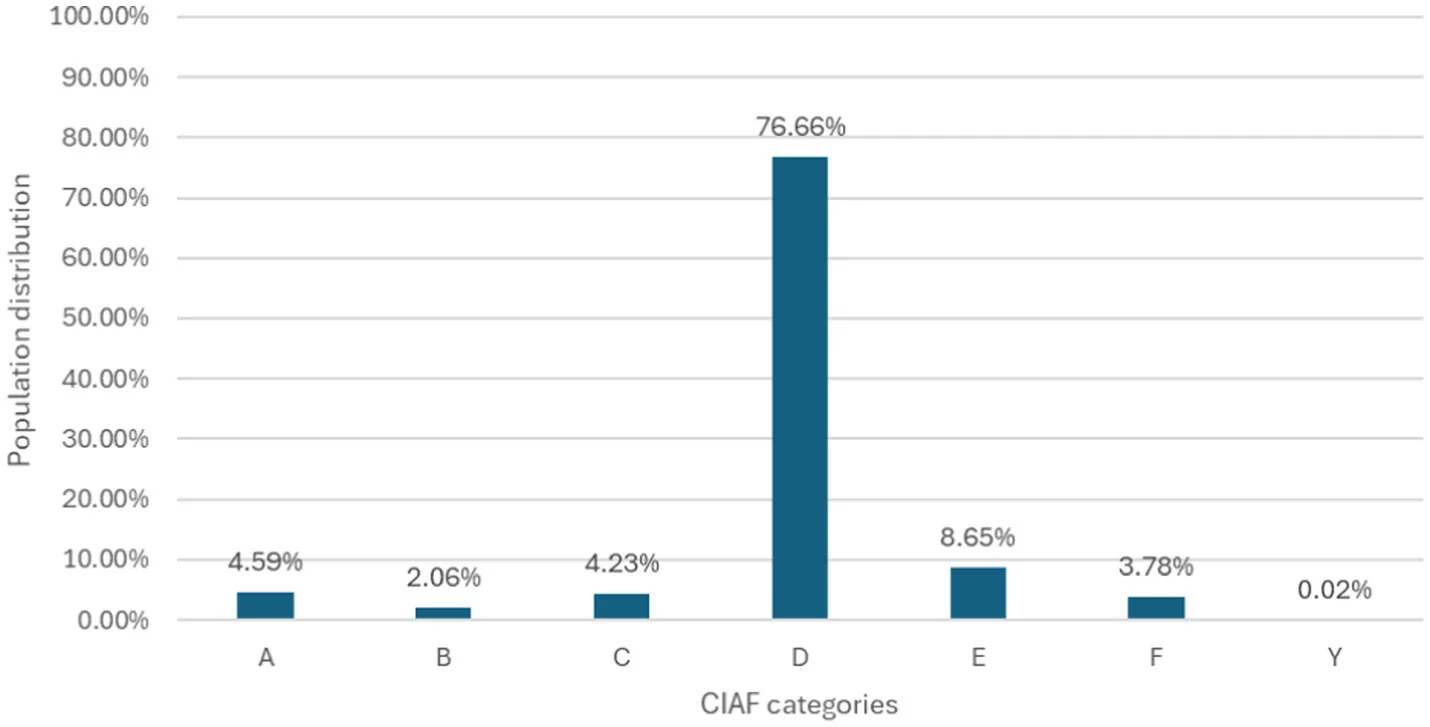
Overall distribution of CIAF categories of children (0-59 m) in Gabon (2019–2021), Gambia (2019–2020), Liberia (2019–2020), Mauritania (2019–2021), and Nigeria (2021).

**Figure 2:**
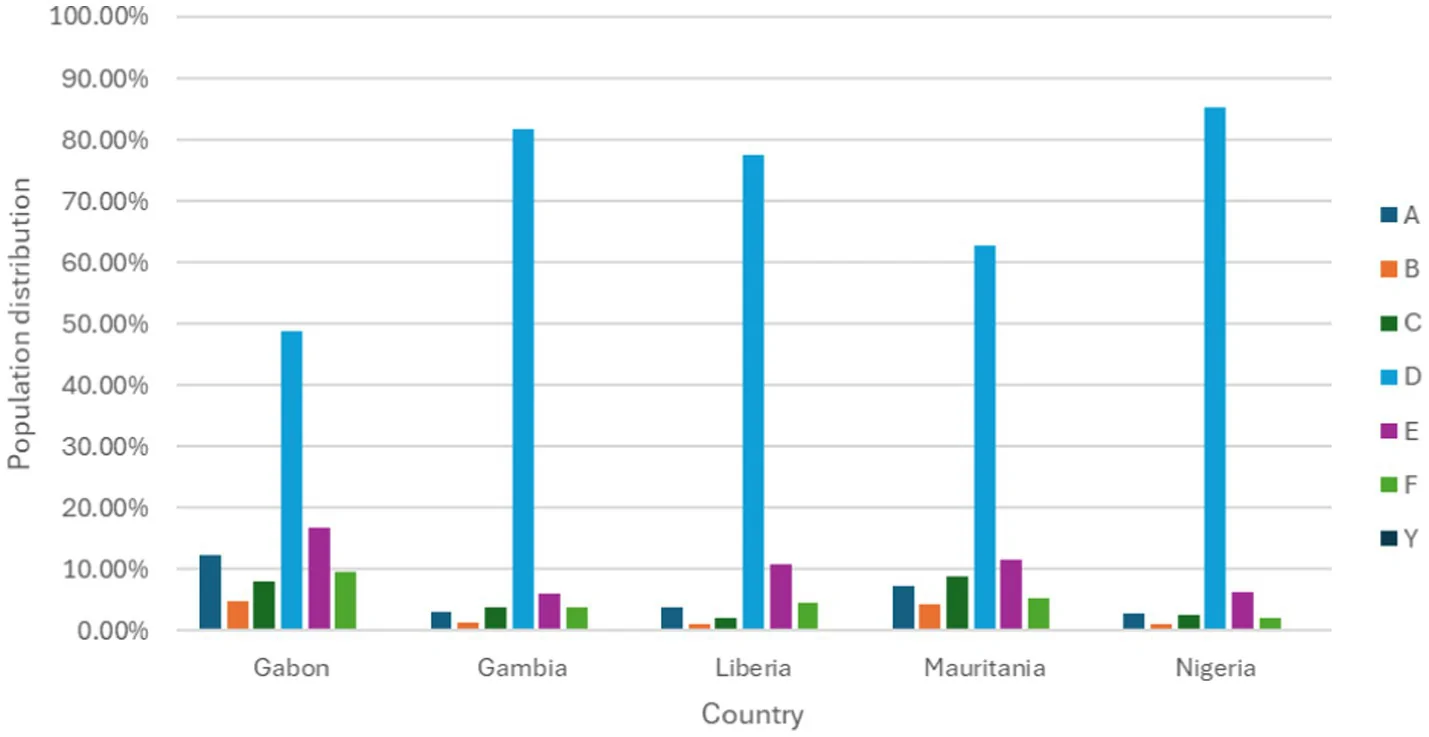
Country level distribution of CIAF categories of children (0-59 m) in Gabon (2019–2021), Gambia (2019–2020), Liberia (2019–2020), Mauritania (2019–2021), and Nigeria (2021).

A child was classified as having moderate-to-severe anemia if their altitude-adjusted hemoglobin concentration was below 10.0 g/dL. Children with an altitude-adjusted hemoglobin concentration of 10.0 g/dL or higher were classified as not having moderate-to-severe anemia and constituted the comparison group. The outcome was binary-coded as 1 for moderate-to-severe anemia and 0 otherwise. Figures 3, 4 display the distribution of Anemia for the country and pooled level data sample.

**Figure 3:**
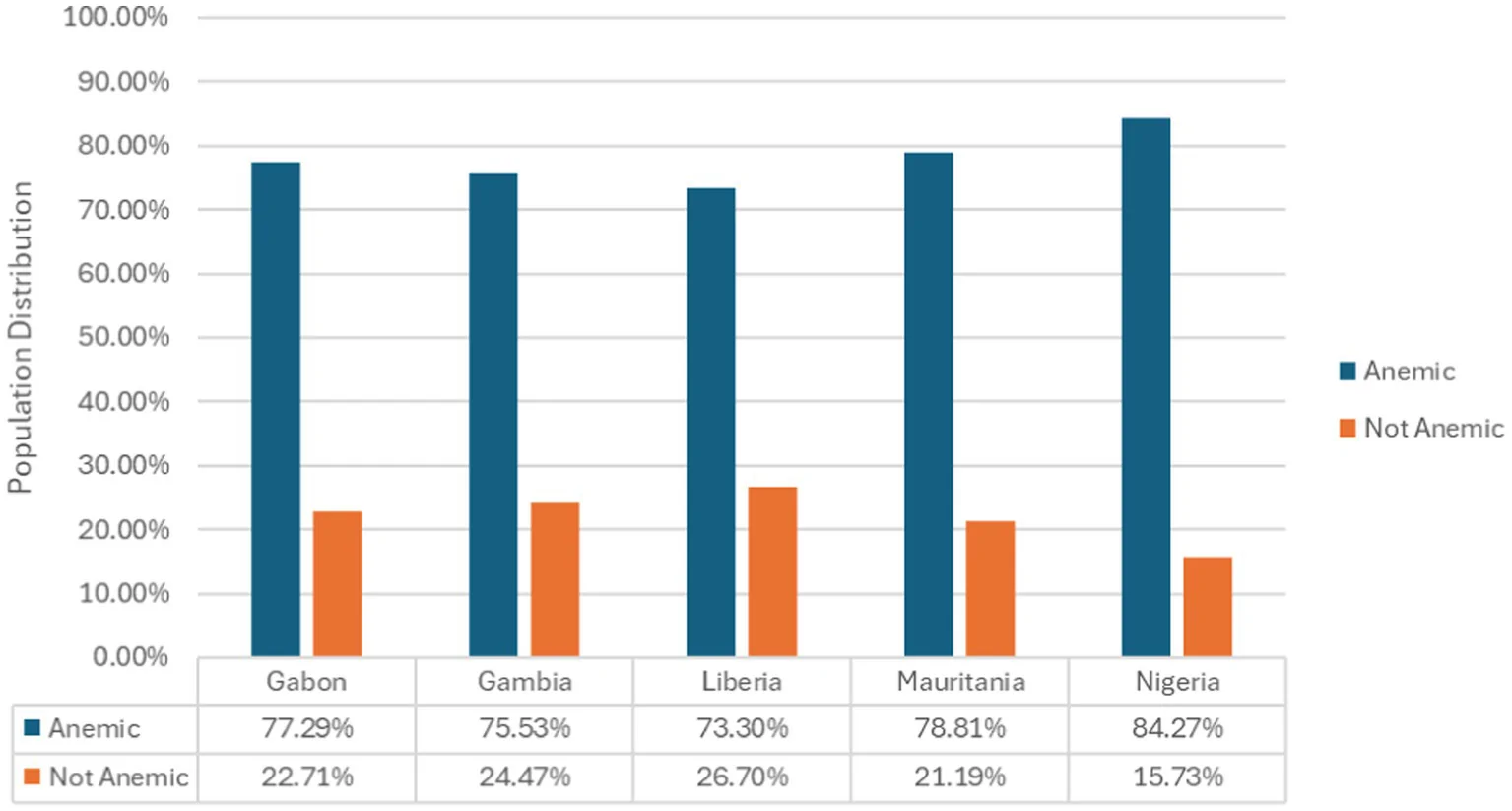
Country level distribution of Anemia of children (0-59 m) in Gabon (2019–2021), Gambia (2019–2020), Liberia (2019–2020), Mauritania (2019–2021), and Nigeria (2021).

**Figure 4:**
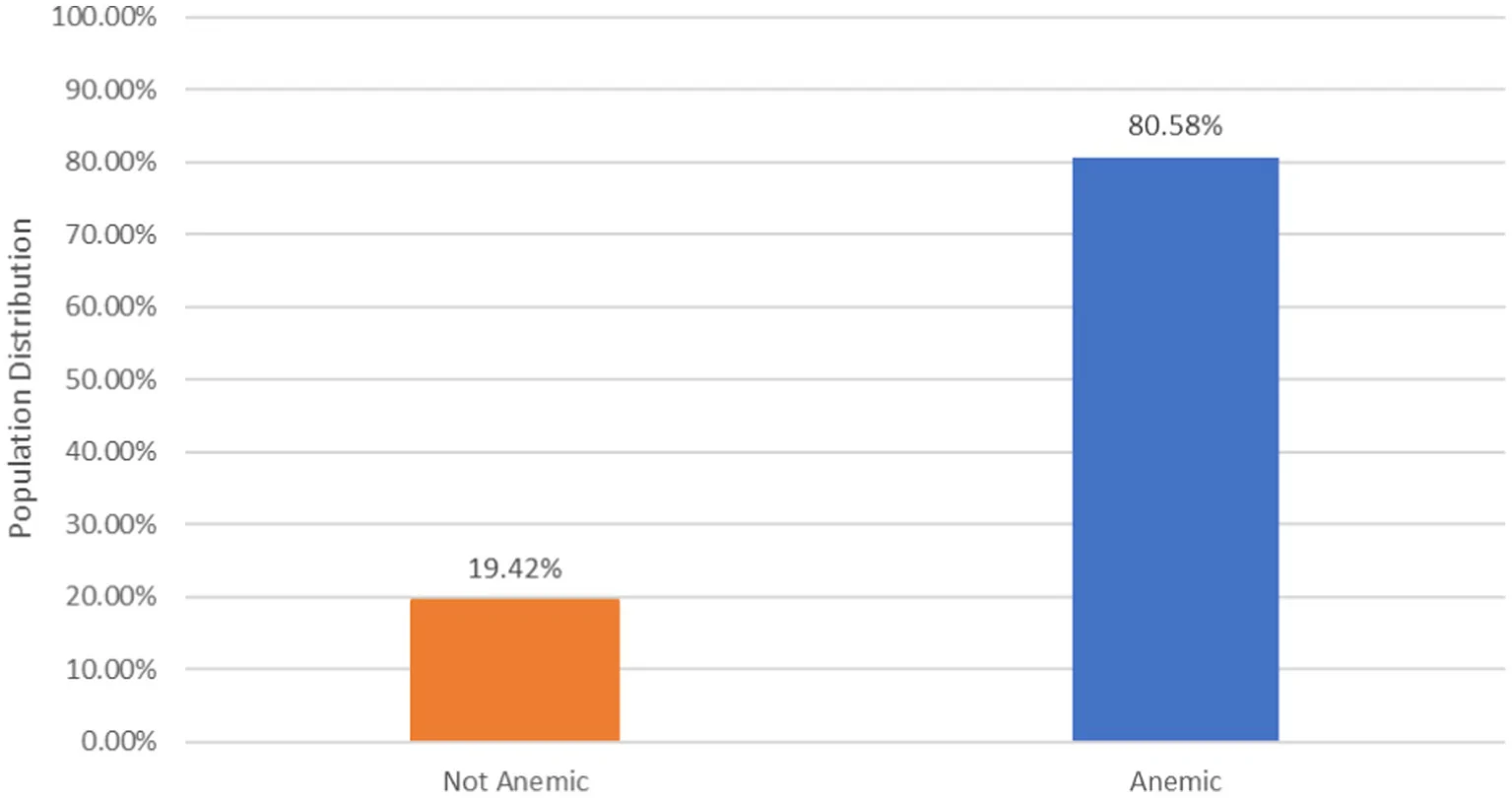
Overall distribution of Anemia of children (0-59 m) in Gabon (2019–2021), Gambia (2019–2020), Liberia (2019–2020), Mauritania (2019–2021), and Nigeria (2021).

Table 2 presents the prevalence rates of anemia and malnutrition across the five selected countries. The findings indicate that anemia remains a critical public health concern, with an overall prevalence of 80.58% among children under five. Similarly, malnutrition was highly prevalent, affecting 95.41% of the sampled population. Furthermore, the co-occurrence of both anemia and malnutrition was observed in 77.55% of children, highlighting the significant overlap between these conditions. These findings underscore the urgent need for integrated nutritional and health interventions to address the dual burden of anemia and malnutrition in the region.

**Table 2:** Characteristics by anemia and malnutrition status.

Target	Nourished	Malnourished	Total
Anaemic	3.02%	77.55%	80.58%
Not anaemic	1.57%	17.85%	19.42%
Total	4.59%	95.41%	100.00%

The CIAF prevalence observed in this study was substantially higher than estimates reported in much of the previous literature. The CIAF outcome was nevertheless retained after verification of the anthropometric scaling, use of the −2 standard-deviation cut-offs, exclusion of missing and biologically implausible measurements, and correct classification of the no-failure group. Because CIAF identifies any occurrence of stunting, wasting, or underweight, it is expected to yield a higher prevalence than each conventional indicator considered separately. However, the unusually high magnitude observed here should be interpreted in relation to the composition of the pooled analytical sample and should not be treated as directly equivalent to country-specific, survey-weighted prevalence estimates.

## Methodology

### Model outline

Traditional regression models often fail to adequately capture the dependence structure between two related health outcomes, such as anemia and malnutrition. A bivariate copula-based approach offers a flexible way to model joint dependencies between these conditions while accommodating non-Gaussian distributions and tail dependencies (19, 20). In addition, geographic disparities in health outcomes necessitate the incorporation of spatial effects, which are efficiently handled using geo-additive modeling (21).

Let $Y_{1i}$ and $Y_{2i}$ denote the two binary outcomes for child i . The marginal probability of each outcome was modelled separately using an additive logistic regression model. For k=1,2 ,

$$Y_{\mathrm{ki}}∼\text{Bernoulli}(p_{\mathrm{ki}}),$$

with

$$\text{logit}(p_{\mathrm{ki}})=\eta_{\mathrm{ki}}=\beta_{0k}+\sum_{j=1}^{J_{k}}\beta_{\mathrm{jk}}X_{\mathrm{jki}}+\sum_{r=1}^{R_{k}}f_{\mathrm{rk}}(Z_{\mathrm{rki}})+f_{\mathrm{sp},k}(s_{i}),k=1,2,$$

where $p_{\mathrm{ki}}=P(Y_{\mathrm{ki}}=1|x_{\mathrm{ki}})$ , $\beta_{0k}$ denotes the intercept for outcome k , $X_{\mathrm{jki}}$ denotes the parametric covariates, $f_{\mathrm{rk}}(\cdot )$ denotes smooth functions of continuous covariates, and $f_{\mathrm{sp},k}(s_{i})$ denotes the spatial effect for the location or region $s_{i}$ . The marginal probabilities are consequently given by

$$p_{\mathrm{ki}}=\frac{\exp(\eta_{\mathrm{ki}})}{1+\exp(\eta_{\mathrm{ki}})},k=1,2.$$

Dependence between the two binary outcomes was introduced through a bivariate copula C(·;·;θ), where θ is the copula dependence parameter. The joint probability that both outcomes equal one is.

$$\pi_{11,i}=P(Y_{1i}=1,Y_{2i}=1|x_{i})=C(p_{1i},p_{2i};\theta ).$$

The remaining joint outcome probabilities are.

$$\pi_{10,i}=P(Y_{1i}=1,Y_{2i}=0|x_{i})=p_{1i}-C(p_{1i},p_{2i};\theta ),$$

$$\pi_{01,i}=P(Y_{1i}=0,Y_{2i}=1|x_{i})=p_{2i}-C(p_{1i},p_{2i};\theta ),$$

and

$$\pi_{00,i}=P(Y_{1i}=0,Y_{2i}=0|x_{i})=1-p_{1i}-p_{2i}+C(p_{1i},p_{2i};\theta ).$$

The model parameters were estimated jointly by maximizing the copula-based log-likelihood:

$$\ell (\beta_{1},\beta_{2},\theta )=\sum_{i=1}^{n}[\begin{array}{l} Y_{1i}Y_{2i}\log(\pi_{11,i})+Y_{1i}(1-Y_{2i})\log(\pi_{10,i})+\\ \left(1-Y_{1i})Y_{2i}\log(\pi_{01,i})+(1-Y_{1i}\right)\\ \left(1-Y_{2i})\log(\pi_{00,i}\right) \end{array}].$$

Where smooth and spatial effects were included, estimation was based on the corresponding penalized joint log-likelihood:

$$\ell_{p}=\ell -\frac{1}{2}\sum_{k=1}^{2}\sum_{r=1}^{R_{k}}\lambda_{\mathrm{rk}}\gamma_{\mathrm{rk}}^{T}S_{\mathrm{rk}}\gamma_{\mathrm{rk}},$$

where $\gamma_{\mathrm{rk}}$ denotes the coefficient vector for a smooth component, $S_{\mathrm{rk}}$ is its penalty matrix, and $\lambda_{\mathrm{rk}}$ is the associated smoothing parameter (22, 23). The model was fitted using the GJRM package in R, with a separate formula specified for each marginal outcome and the copula used to represent residual dependence between the outcomes.

This presentation reflects GJRM’s general penalized maximum-likelihood framework, in which smooth effects are represented using penalized regression splines and the marginal outcomes are connected by a copula distribution.

### Copula selection

The process of selecting an appropriate copula family and accurately estimating its parameters is a critical step in modeling multivariate dependence structures. A well-fitted copula ensures that both the dependence structure—captured by the copula family—and the dependence strength—quantified by its parameters—are properly represented (24).

Archimedean copulas represent a widely used class of copula functions, particularly valued for their simplicity, flexibility, and ability to model a broad range of dependence structures. These copulas are defined through a generator function, which allows for the construction of bivariate and multivariate copulas with a single parameter governing the strength of dependence. Their mathematical tractability and associative property make them especially suitable for high-dimensional modeling and simulation tasks (25).

Archimedean copulas such as Clayton, Gumbel, and Frank are popular choices due to their ability to capture tail dependencies—a critical aspect when modeling extreme events. For instance, the Clayton copula is known for its lower tail dependence, making it suitable for modeling joint extreme losses, while the Gumbel copula captures upper tail dependence, useful in scenarios involving joint extreme gains. Despite their relatively simple structure, Archimedean copulas offer robust performance in capturing nonlinear dependencies, which are often overlooked by traditional correlation-based methods (25).

Archimedean copulas are defined using a generator function λ(t) with the following properties (2):

λ(1)=0

$$\lambda^{\prime }(t)\leq 0\;\text{for}\;\mathrm{all}\;t\in (0,1)$$

$$\lambda^{\prime \prime }(t)\geq 0\;\text{for}\;\mathrm{all}\;t\in (0,1)$$

Now let $\lambda^{\left(-1\right)}$ denote the pseudo-inverse of the generator function λ . This pseudo-inverse behaves like the standard inverse for values t∈[0,λ(0)] and is defined to be zero for t≥λ(0) . Using this formulation, the Archimedean copula for two variables u and v is expressed as:

$$C(u,v)=\lambda^{\left(-1\right)}(\lambda (u)+\lambda (v))$$

Archimedean copulas possess two key structural properties:

- Symmetry: C(u,v)=C(v,u) , meaning the copula treats both variables equally.
- Associativity: C(C(u,v),w)=C(u,C(v,w)) , which allows for consistent extension to higher dimensions.

These properties make Archimedean copulas particularly useful in multivariate modeling, especially when the dependence structure is complex but needs to be captured with a single parameter.

The copulas that are investigated within this paper are outlined below:

#### Elliptical copula

- Normal Copula
$$C_{\text{Normal}}(u_{1};u_{2};p)=\Phi_{p}(\Phi^{-1}(u_{1}),\Phi^{-1}(u_{2}))$$

Where $\Phi^{-1}$ is the inverse standard normal CDF, and $\Phi_{p}$ is the bivariate normal CDF with correlation p and $u_{1}$ and $u_{2}$ are the CDF of variable Y and X (26).

- T Copula
$$C_{t}(u_{1},u_{2};p,v)=t_{p}{,}_{v}(t_{v}^{-1}(u_{1}),t_{v}^{-1}(u_{2}))$$

Where $t_{v}^{-1}$ is the inverse CDF of the univariate t-distribution with v degrees of freedom, and $t_{p}{,}_{v}$ is the bivariate t-distribution CDF (27).

#### Archimedean copula

- Clayton copula
$$C_{\text{Clayton}}(u_{1},u_{2};\theta )={\left(u_{1}^{-\theta }+u_{2}^{-\theta }-1\right)}^{-1/\theta },\theta >0$$

Where $u_{1}$ and $u_{2}$ are the CDF of variable Y and X (28).

- Gumbel copula
$$C_{\text{Gumbel}}(u_{1},u_{2};\theta )=\exp[-{\left(\ln(u_{1})\right)}^{\theta }+{\left(-\ln{\left(u_{2}\right)}^{\theta }\right)}^{\frac{1}{\theta }}],\theta \geq 1$$

Where $u_{1}$ and $u_{2}$ are the CDF of variable Y and X (29).

- Frank Copula
$$C_{\text{Frank}}(u_{1},u_{2};\theta )=-\frac{1}{\theta }\;\ln(1+\frac{\left(e^{-\theta u_{1}}-1)(e^{-\theta u_{2}}-1\right)}{e^{-\theta }-1}),\theta \neq 0$$

Where $u_{1}$ and $u_{2}$ are the CDF of variable Y and X (30).

#### Visual comparison of copula dependence structures

The following contour plots (Figure 5) illustrate the dependence structures modeled by five different copulas using simulated data for illustrative purposes. These visualizations help in understanding how each copula captures relationships between variables, especially in the tails of the distribution.

- Normal Copula: Exhibits symmetric dependence with no tail dependence. It assumes linear correlation and is suitable for modeling moderate relationships.
- T Copula: Similar to the Normal copula but with tail dependence in both upper and lower tails. Useful for modeling extreme co-movements.
- Clayton Copula: Captures strong lower tail dependence, making it suitable for scenarios where joint extreme losses are of interest.
- Gumbel Copula: Captures strong upper tail dependence, ideal for modeling joint extreme gains or risks in the upper tail.
- Frank Copula: Symmetric with no tail dependence. It provides flexibility in modeling moderate dependence across the distribution.

**Figure 5:**
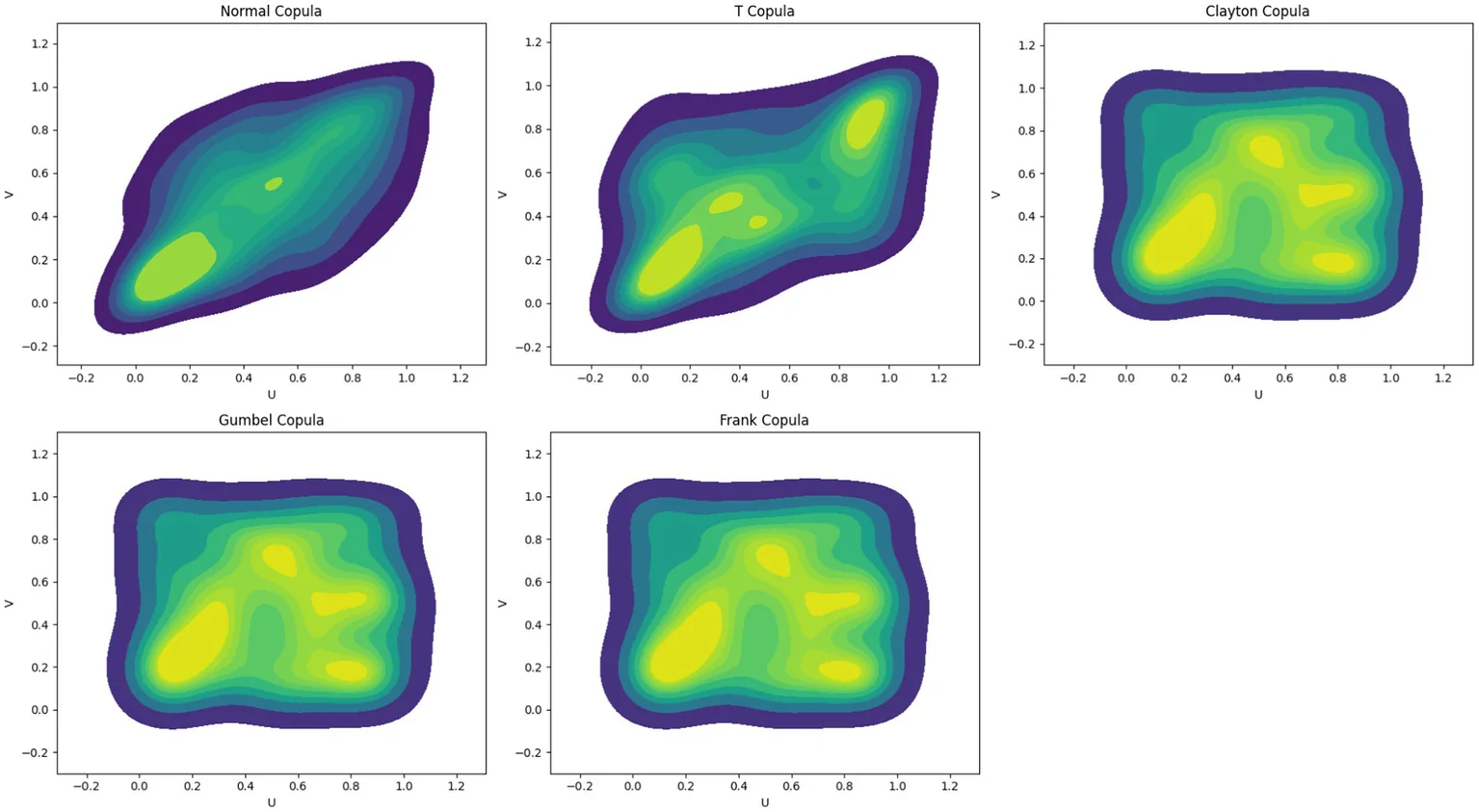
Contour Plots using simulated data.

The bivariate copula geo-additive model provides a robust framework for jointly analyzing anemia and malnutrition while accounting for spatial heterogeneity and complex dependencies. This approach enhances the understanding of underlying risk factors and informs targeted public health interventions in West Africa.

## Results

### Copula and link function selection

Model specification involved two key selection steps: selecting the most appropriate copula family to capture dependence and identifying the most suitable link function for the marginal models. In both cases, alternative specifications were compared using the maximum likelihood (ML) method. Under the same dataset and estimation framework, the model yielding the highest maximized log-likelihood value was selected, as this criterion reflects the specification that best aligns the predicted distribution with the observed data (19, 31).

For the dependence structure (Table 3), five copula families were evaluated: Normal [19.04], t [21.36], Clayton [5.06], Gumbel [28.01], and Frank [11.10]. The Gumbel copula achieved the highest log-likelihood (28.01) and lowest AIC (183.98), with α = 1.03 (SE = 0.005), capturing the asymmetric tail dependence relevant to the research context. The upper-tail dependence represented by the Gumbel copula corresponds to a greater tendency for the two conditions to occur jointly among children with higher underlying probabilities of both outcomes (20).

**Table 3:** The Log likelihood results of the copula selection.

Copula	Log likelihood	AIC
Normal copula	19.04	201.92
T copula	21.36	197.28
Clayton copula	5.064	229.87
Gumbel copula	28.01	183.98
Frank copula	11.1	217.80

For the marginal models (Table 4), four link function combinations were compared: (“logit,”“logit”) [−14,010.62], (“logit,”“cloglog”) [−14,015.80], (“logit,”“probit”) [−14,011.46], and (“cloglog,”“probit”) [−14,001.99]. The combination (“cloglog,”“probit”) achieved the highest log-likelihood (−14,001.99), indicating the best fit among candidates, with parameter estimates demonstrating high precision and successful convergence (32).

**Table 4:** The Log likelihood results of the link selection.

Link Selection	Loglik
c(“logit,”“logit”)	−14,010.62
c(“logit,”“cloglog”)	−14,015.8
c(“logit,”“probit”)	−14,011.46
c(“cloglog,”“probit”)	−14,001.99

Together, these selection procedures ensured that both the marginal and joint components of the model were specified to achieve the best possible empirical fit within the study framework.

### Fixed effects results

The results of the fixed effects are based on the Gumbel copula and c (“cloglog,” “probit”) link function. The package GJRM (Generalized Joint Regression Modelling), v3.63, was used to obtain the results (41).

The results of the Generalized Joint Regression Model (GJRM) examining predictors of malnutrition and anemia are presented in Table 5. Several key factors were significantly associated with the risk of malnutrition and anemia among children.

**Table 5:** Parameter estimates of the fixed effects for the bivariate copula regression model of malnutrition and anemia.

Variable	Malnutition		Anemia	
	Est (95% CI)	Pr(>|*z*|)	Est (95% CI)	Pr(>|*z*|)
Residence_type (Ref = Rural)				
Urban	0.029 (−0.032, 0.089)	0.3518	−0.106 (−0.160, −0.052)	0.0001
Mother_highest_education (Ref = Higher)				
No Education	0.132 (0.016, 0.247)	0.0255	0.188 (0.079, 0.296)	0.0007
Primary	0.187 (0.073, 0.301)	0.0013	0.117 (0.010, 0.225)	0.0324
Secondary	0.075 (−0.025, 0.175)	0.1432	0.044 (−0.052, 0.141)	0.3709
Source of drinking water (Ref = Bottle/Sachet)				
Piped	0.388 (0.105, 0.671)	0.0072	0.254 (−0.032, 0.540)	0.0818
Natural Sources	0.348 (0.057, 0.639)	0.0191	0.110 (−0.184, 0.403)	0.4647
Tanker	0.541 (0.209, 0.872)	0.0014	0.049 (−0.273, 0.371)	0.7644
Well	0.414 (0.129, 0.700)	0.0045	0.213 (−0.076, 0.502)	0.1482
Other	0.232 (−0.071, 0.535)	0.1336	0.313 (0.006, 0.620)	0.0460
Type of toilet facility (Ref = Flush)				
Pit	0.056 (−0.018, 0.129)	0.1398	0.025 (−0.043, 0.094)	0.4720
Other	0.074 (−0.009, 0.158)	0.0815	0.0004 (−0.077, 0.078)	0.9912
Household has electricity (Ref = No)				
Yes	0.052 (−0.016, 0.120)	0.1324	−0.006 (−0.067, 0.055)	0.8494
Gender of the head of the household (Ref = Female)				
Male	0.007 (−0.053, 0.068)	0.8175	−0.008 (−0.066, 0.050)	0.7821
Type of cooking fuel (Ref = Electricity)				
Wood	0.084 (−0.176, 0.343)	0.5272	0.137 (−0.110, 0.384)	0.2780
Gas	0.045 (−0.215, 0.304)	0.7357	0.045 (−0.203, 0.293)	0.7240
No food cooked	−0.223 (−1.038, 0.591)	0.5907	−0.744 (−1.636, 0.149)	0.1024
Wealth Index (Ref = Middle)				
Poorest	0.028 (−0.055, 0.112)	0.5056	0.096 (0.021, 0.171)	0.0124
Poorer	0.016 (−0.061, 0.092)	0.6883	0.044 (−0.025, 0.113)	0.2084
Richer	−0.062 (−0.142, 0.019)	0.1336	−0.026 (−0.100, 0.047)	0.4835
Richest	−0.152 (−0.258, −0.046)	0.0049	−0.182 (−0.280, −0.084)	0.0003
Slept under a net last night (Ref = Both treated and untreated)				
No net	1.437 (−0.550, 3.424)	0.1564	0.330 (−1.464, 2.123)	0.7188
Only treated nets	1.434 (−0.553, 3.421)	0.1572	0.236 (−1.557, 2.030)	0.7960
Only untreated nets	1.401 (−0.589, 3.392)	0.1677	0.222 (−1.575, 2.019)	0.8089
Visted a health facility in the last 12 months (Ref = No)				
Yes	−0.018 (−0.070, 0.035)	0.5110	0.014 (−0.034, 0.061)	0.5718
Gave the child milk (Ref = No)				
Yes	0.028 (−0.037, 0.094)	0.3950	0.006 (−0.053, 0.066)	0.8380
Gave the child formula (Ref = No)				
Yes	0.027 (−0.056, 0.109)	0.5296	0.001 (−0.078, 0.081)	0.9720
Gave the child fortified food (Ref = No)				
Yes	−0.089 (−0.172, −0.005)	0.0383	−0.080 (−0.159, −0.001)	0.0472
Gave the child tubers (Ref = No)				
Yes	−0.020 (−0.087, 0.046)	0.5517	−0.010 (−0.069, 0.049)	0.7395
Gave the child eggs (Ref = No)				
Yes	−0.055 (−0.135, 0.025)	0.1762	−0.148 (−0.220, −0.075)	<0.0001
Gave the child meat (Ref = No)				
Yes	−0.036 (−0.109, 0.037)	<0.0001	0.053 (−0.013, 0.120)	0.1147
Has a mosquito net (Ref = No)				
Yes	0.011 (−0.059, 0.081)	0.7555	0.030 (−0.034, 0.094)	0.3597
Gender of the child (Ref = Female)				
Male	0.096 (0.050, 0.141)	<0.0001	−0.0001 (−0.041, 0.041)	0.9970
Fever in the last 2 weeks (Ref = Yes)				
No	0.084 (0.022, 0.145)	<0.0001	0.029 (−0.026, 0.083)	0.3030
Cough in the last 2 weeks (Ref = Yes)				
No	0.023 (−0.041, 0.088)	0.4830	−0.012 (−0.070, 0.046)	0.6852
Weight category (Ref = Extremely low birth weight < 1 kg)				
Very low birth weight: < 1.5 kg	−0.108 (−0.301, 0.085)	0.2732	−0.289 (−0.475, −0.104)	0.0023
Low birth weight: < 2.5 kg	0.058 (−0.090, 0.205)	0.4359	−0.146 (−0.295, 0.004)	0.9992
Normal birth weight > 2.5 kg	0.460 (0.256, 0.664)	<0.0001	1.826 (1.523, 2.129)	<0.0001

Residence type was significantly associated with anemia risk: children residing in urban areas had a lower risk of anemia compared to those in rural areas (Estimate = −0.1060, *p* = 0.0001), while no significant association was found for malnutrition (*p* = 0.3518).

Maternal education was a strong predictor for both outcomes. Children whose mothers had no education (Estimate = 0.1316, *p* = 0.0255) or only primary education (Estimate = 0.1871, *p* = 0.0013) had significantly higher malnutrition risk compared to those whose mothers had higher education. Similarly, for anemia, children of mothers with no education (Estimate = 0.1875, *p* = 0.0007) or primary education (Estimate = 0.1175, *p* = 0.0324) were at higher risk.

Source of drinking water was significantly associated with malnutrition. Compared to bottled/sachet water, piped (Estimate = 0.3881, *p* = 0.0072), natural sources (Estimate = 0.3482, *p* = 0.0191), tanker (Estimate = 0.5406, *p* = 0.0014), and well water (Estimate = 0.4142, *p* = 0.0045) were all linked to increased malnutrition risk. For anemia, only the “Other” water source category showed a significant association (Estimate = 0.3129, *p* = 0.0460).

Wealth index was inversely associated with both outcomes. Children from the richest households had significantly lower risks of malnutrition (Estimate = −0.1519, *p* = 0.0049) and anemia (Estimate = −0.1818, *p* = 0.0003) compared to those from middle-income households. Children from the poorest households had a higher anemia risk (Estimate = 0.0960, *p* = 0.0124), though no significant association was found for malnutrition.

Child feeding practices also influenced outcomes. Children who were given fortified food had a lower risk of both malnutrition (Estimate = −0.0886, *p* = 0.0383) and anemia (Estimate = −0.0798, *p* = 0.0472). Egg consumption was significantly protective against anemia (Estimate = −0.1476, *p* < 0.0001), though not significantly associated with malnutrition. Other dietary variables such as milk, formula, tubers, and meat did not show significant associations.

Child gender was significantly associated with malnutrition, with male children at higher risk (Estimate = 0.0955, *p* < 0.0001), but showed no significant association with anemia. Children who had not experienced fever during the 2 weeks preceding the survey had a higher malnutrition linear predictor than children for whom fever was reported (Estimate = 0.084, *p* < 0.0001). By contrast, the absence of cough during the same period was not significantly associated with malnutrition (Estimate = 0.023, *p* = 0.483. These findings represent adjusted cross-sectional associations and do not establish a protective effect of recent fever or a causal relationship between recent symptoms and malnutrition.

Birth weight category was a strong predictor. Compared to children with extremely low birth weight, those with normal birth weight had significantly lower risk of malnutrition (Estimate = 0.4599, *p* < 0.0001) and anemia (Estimate = 1.8260, *p* < 0.0001). Very low birth weight was significantly associated with reduced anemia risk (Estimate = −0.2894, *p* = 0.0023), but not with malnutrition.

Other variables such as toilet facility type, household electricity, gender of household head, cooking fuel type, mosquito net usage, and health facility visits did not show consistent or significant associations with either outcome. The estimated malnutrition coefficients for the mosquito-net-use categories were positive but imprecise. The wide confidence intervals indicate substantial uncertainty, and no statistically supported association was observed.

The results for the smooth terms in the Generalized Joint Regression Model (GJRM) indicated several significant non-linear associations with the outcomes of malnutrition and anemia (For malnutrition, spatial variation across geographic coordinates (longitude and latitude) remained a significant predictor (edf = 24.14, *χ*^2^ = 58.24, *p* = 0.0004), indicating notable regional differences in malnutrition risk. Child age in months showed a strong and highly significant non-linear relationship with malnutrition (edf = 6.07, *χ*^2^ = 501.99, *p* < 0.0001), suggesting that age-related growth patterns are critical in determining malnutrition risk. Additionally, mother’s current age exhibited a significant non-linear effect (edf = 1.00, *χ*^2^ = 9.84, *p* = 0.0017), indicating that even a linear smooth term for maternal age contributes meaningfully to malnutrition outcomes. In contrast, the age group of the head of household did not show a significant association (edf = 1.00, *χ*^2^ = 0.088, *p* = 0.7671).

In the model for anemia, spatial location demonstrated an even stronger association (edf = 27.16, *χ*^2^ = 349.67, *p* < 0.0001), highlighting substantial geographic disparities in anemia prevalence. Child age in months was again significantly associated with anemia (edf = 6.17, *χ*^2^ = 28.31, *p* = 0.0003), reinforcing its importance across both outcomes. A non-linear effect of mother’s current age was also observed (edf = 5.82, *χ*^2^ = 22.94, *p* = 0.0017), suggesting a complex relationship between maternal age and anemia risk. While the age of the head of household showed some degree of non-linearity (edf = 6.65), the association with anemia did not reach statistical significance (*χ*^2^ = 13.09, *p* = 0.0737).

These findings underscore the importance of accounting for spatial and non-linear effects in modeling child health outcomes. The interpretation focuses on smooth terms that demonstrated statistically significant associations, ensuring clarity and emphasizing the most influential predictors identified by the model (Table 6).

**Table 6:** Approximate significance of smooth terms.

Target Variable	Malnutrition				Anemia			
	edf	DF	Chi sq	*p*-value	edf	DF	Chi sq	*p*-value
s(longitude,latitude)	24.1400	27.4900	58.2420	0.0004	27.1630	28.7630	349.6700	<0.0001
s(Age_HoH_grp)	1.0000	1.0000	0.0880	0.7671	6.6500	7.6450	13.0900	0.0737
s(Mother_current_age)	1.0000	1.0000	9.8420	0.0017	5.8220	6.9320	22.9400	0.0017
s(Current_Age_child_months)	6.0700	7.2120	501.9940	<0.0001	6.1740	7.3190	28.3100	0.0003

For malnutrition, spatial variation across geographic coordinates (longitude and latitude) remained a significant predictor (edf = 24.14, *χ*^2^ = 58.24, *p* = 0.0004), indicating notable regional differences in malnutrition risk. Child age in months showed a strong and highly significant non-linear relationship with malnutrition (edf = 6.07, *χ*^2^ = 501.99, *p* < 0.0001), suggesting that age-related growth patterns are critical in determining malnutrition risk. Additionally, mother’s current age exhibited a significant non-linear effect (edf = 1.00, *χ*^2^ = 9.84, *p* = 0.0017), indicating that even a linear smooth term for maternal age contributes meaningfully to malnutrition outcomes. In contrast, the age group of the head of household did not show a significant association (edf = 1.00, *χ*^2^ = 0.088, *p* = 0.7671).

These findings underscore the importance of accounting for spatial and non-linear effects in modeling child health outcomes. The interpretation focuses on smooth terms that demonstrated statistically significant associations, ensuring clarity and emphasizing the most influential predictors identified by the model.

Figure 6 presents the smooth effects of spatial location, maternal age, and child age on malnutrition. The spatial term, s(longitude,latitude) , indicates substantial geographic variation, with complex curvature and pronounced deviations from the baseline in several locations. The confidence bands exclude the horizontal axis in parts of the study area, indicating statistically significant spatial effects. Overall, the spatial term was significant ( edf=24.14 , $\chi^{2}=58.24$ , df=27.49 , p=0.0004 ), demonstrating that location contributed meaningfully to variation in malnutrition.

**Figure 6:**
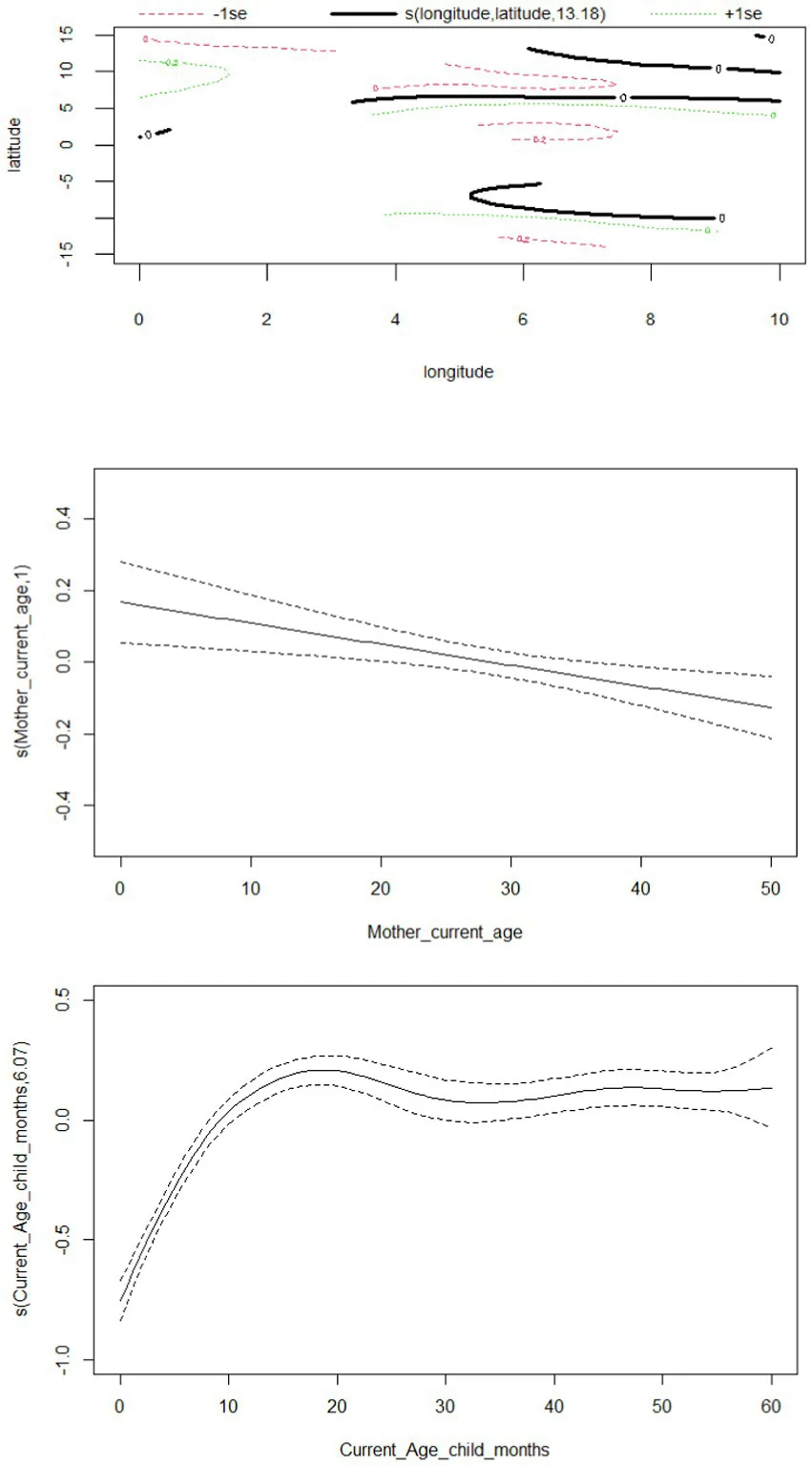
Visualisation of approximate significance of smooth terms for Malnutrition.

Maternal age was negatively associated with malnutrition, with the estimated smooth displaying a modest downward trend across the observed age range. This pattern suggests that the malnutrition linear predictor generally decreased as maternal age increased, particularly between approximately 20 and 40 years. Although maternal age was specified as a smooth term, its effective degrees of freedom of 1.00 indicate that the estimated relationship was essentially linear. The effect was statistically significant ( edf=1.00 , $\chi^{2}=9.84$ , df=1.00 , p=0.0017 ).

Child age showed a strong nonlinear association with malnutrition. The estimated smooth changed markedly during the first 24 months of life and subsequently became more gradual through 60 months. The confidence bands exclude the horizontal axis over portions of the age range, particularly during early childhood, indicating that the association differed significantly from the baseline in those periods. Child age was the most prominent smooth effect in the malnutrition equation ( edf=6.07 , $\chi^{2}=501.99$ , df=7.21 , p<0.0001 ), highlighting substantial age-related variation in malnutrition during the first 5 years of life.

Figure 7 presents the corresponding smooth effects on anemia. The spatial surface, s(longitude,latitude) , shows marked geographic heterogeneity, including steep gradients and localized areas of higher and lower values of the anemia linear predictor. The spatial effect was highly significant ( edf=27.16 , $\chi^{2}=349.67$ , df=28.76 , p<0.0001 ), indicating that anemia varied considerably across the study area after adjustment for the included covariates.

**Figure 7:**
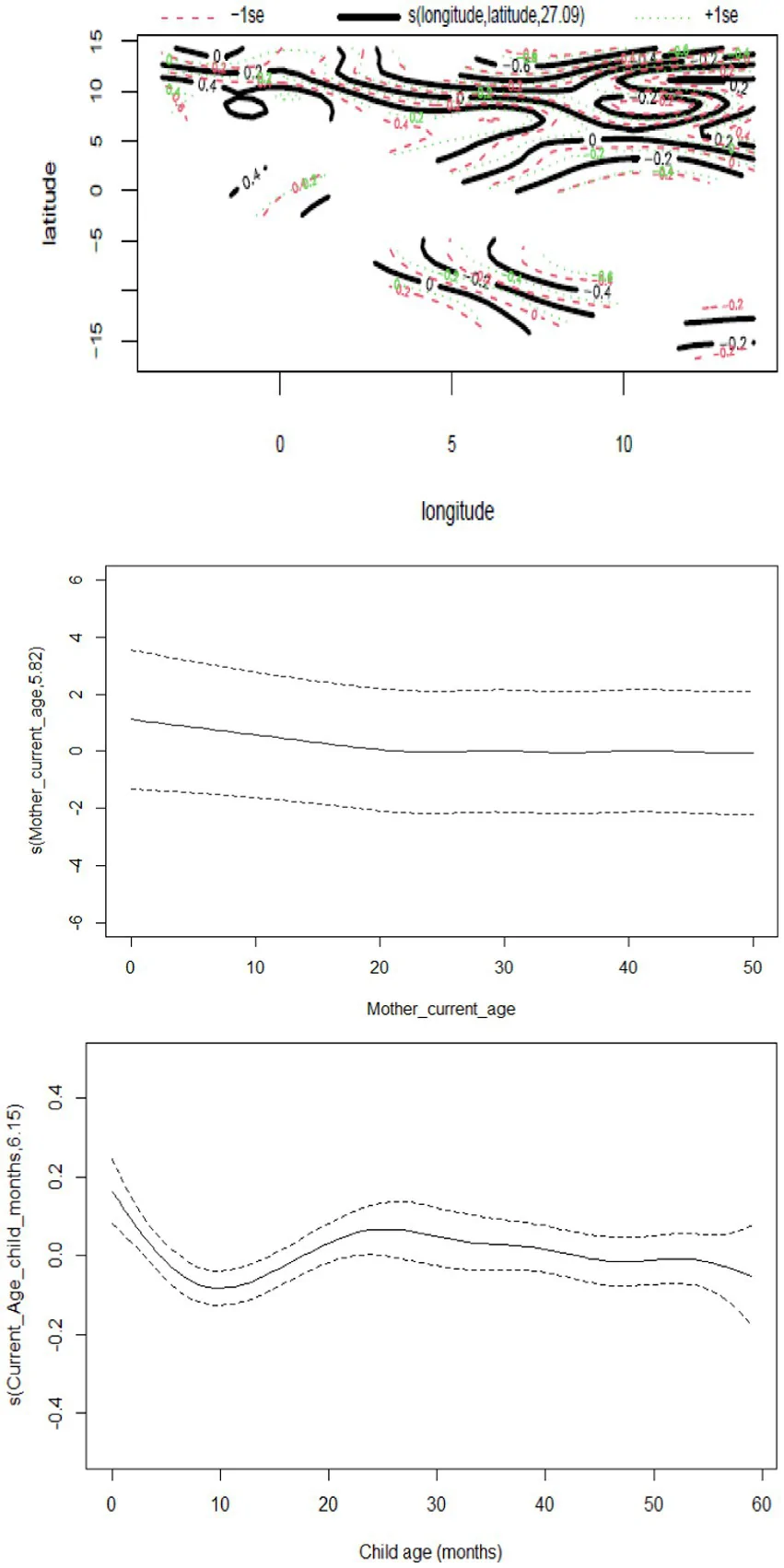
Visualisation of approximate significance of smooth terms for anemia.

In contrast to the approximately linear maternal-age pattern observed for malnutrition, maternal age had a nonlinear association with anemia. The estimated smooth fluctuated across the maternal age range, with the most notable departures from the baseline occurring between approximately 20 and 40 years. This effect was statistically significant ( edf=5.82 , $\chi^{2}=22.94$ , df=6.93 , p=0.0017 ), suggesting that the relationship between maternal age and the anemia linear predictor was non-monotonic.

Child age was also nonlinearly associated with anemia. The fitted curve showed moderate fluctuations across the period from birth to 60 months, indicating that the anemia linear predictor did not follow a simple increasing or decreasing trajectory as children aged. Statistically meaningful departures from the baseline were evident during parts of early childhood. The overall child-age effect was significant ( edf=6.17 , $\chi^{2}=28.31$ , df=7.32 , p=0.0003 ), confirming age-related variation in anemia during the first 5 years of life.

### Estimated joint probability of malnourishment and anemia

The model employed within this study estimated the joint probability (P11) of a child being simultaneously malnourished and anaemic across five West and Central African countries. The spatial distributions are presented in Figures 8-12, with each map illustrating regional variations in joint probabilities using color gradients.

**Figure 8:**
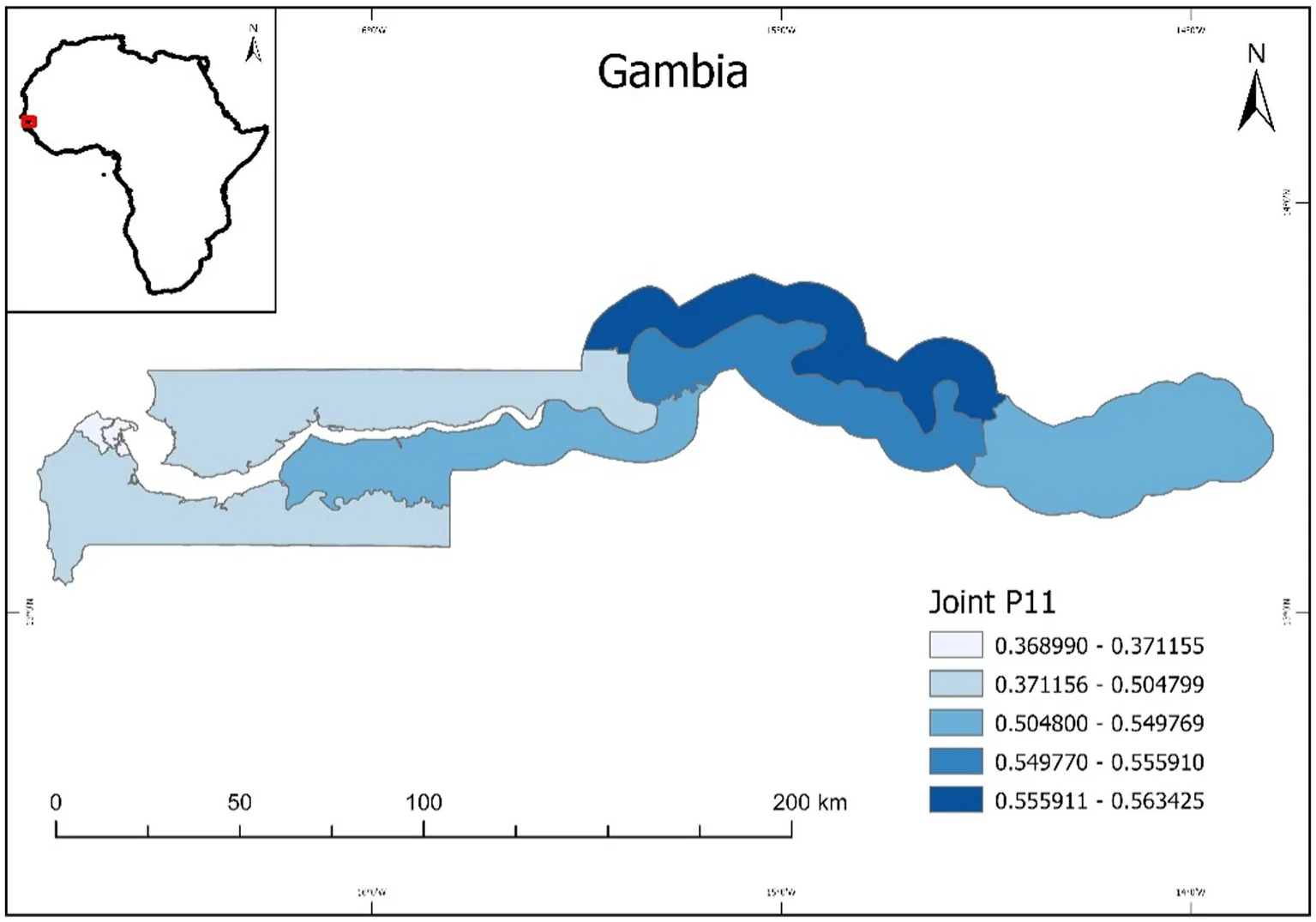
Gambia—joint probability of a child being malnourished and anaemic.

**Figure 9:**
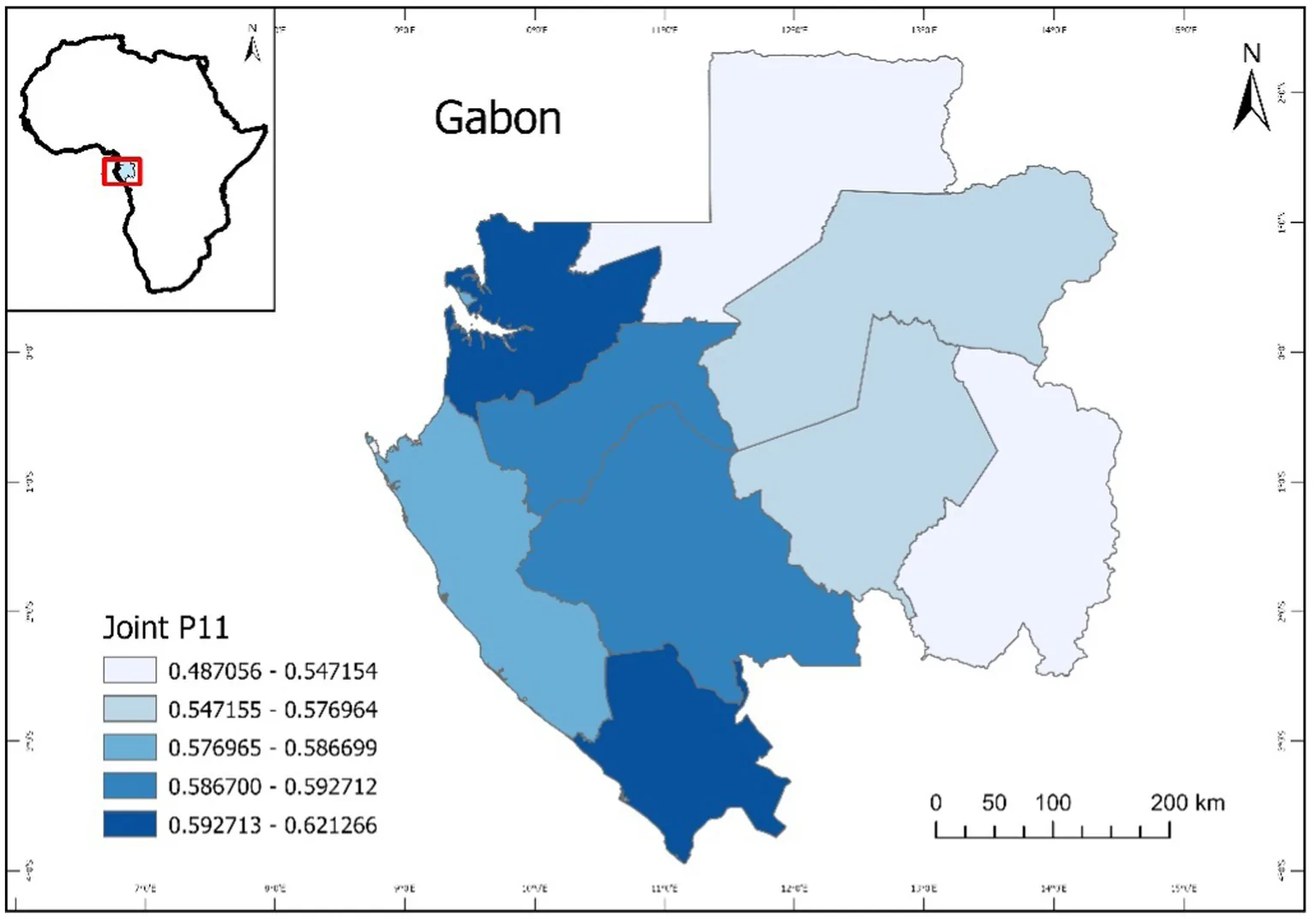
Gabon—joint probability of a child being malnourished and anaemic.

**Figure 10:**
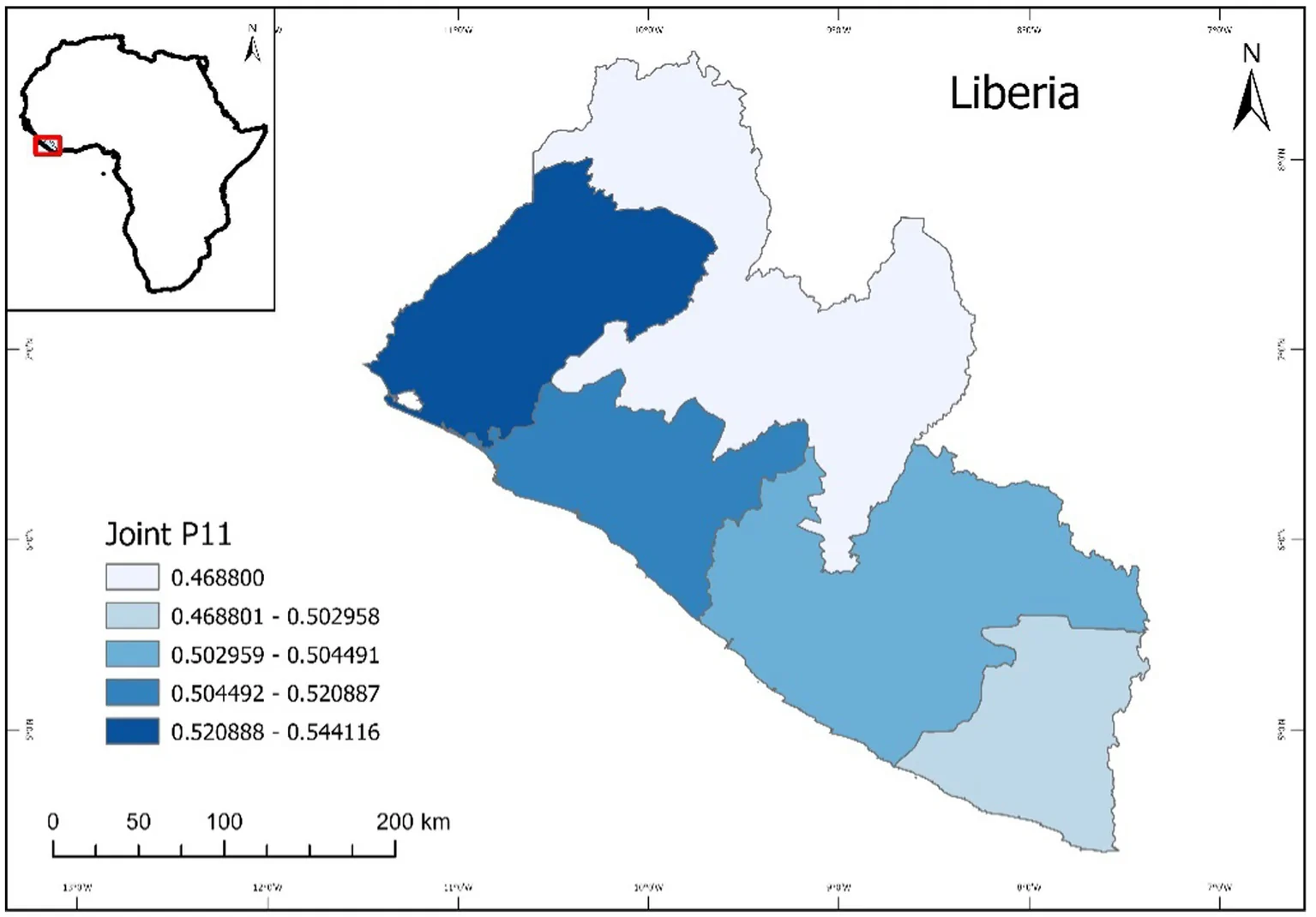
Liberia—joint probability of a child being malnourished and anaemic.

**Figure 11:**
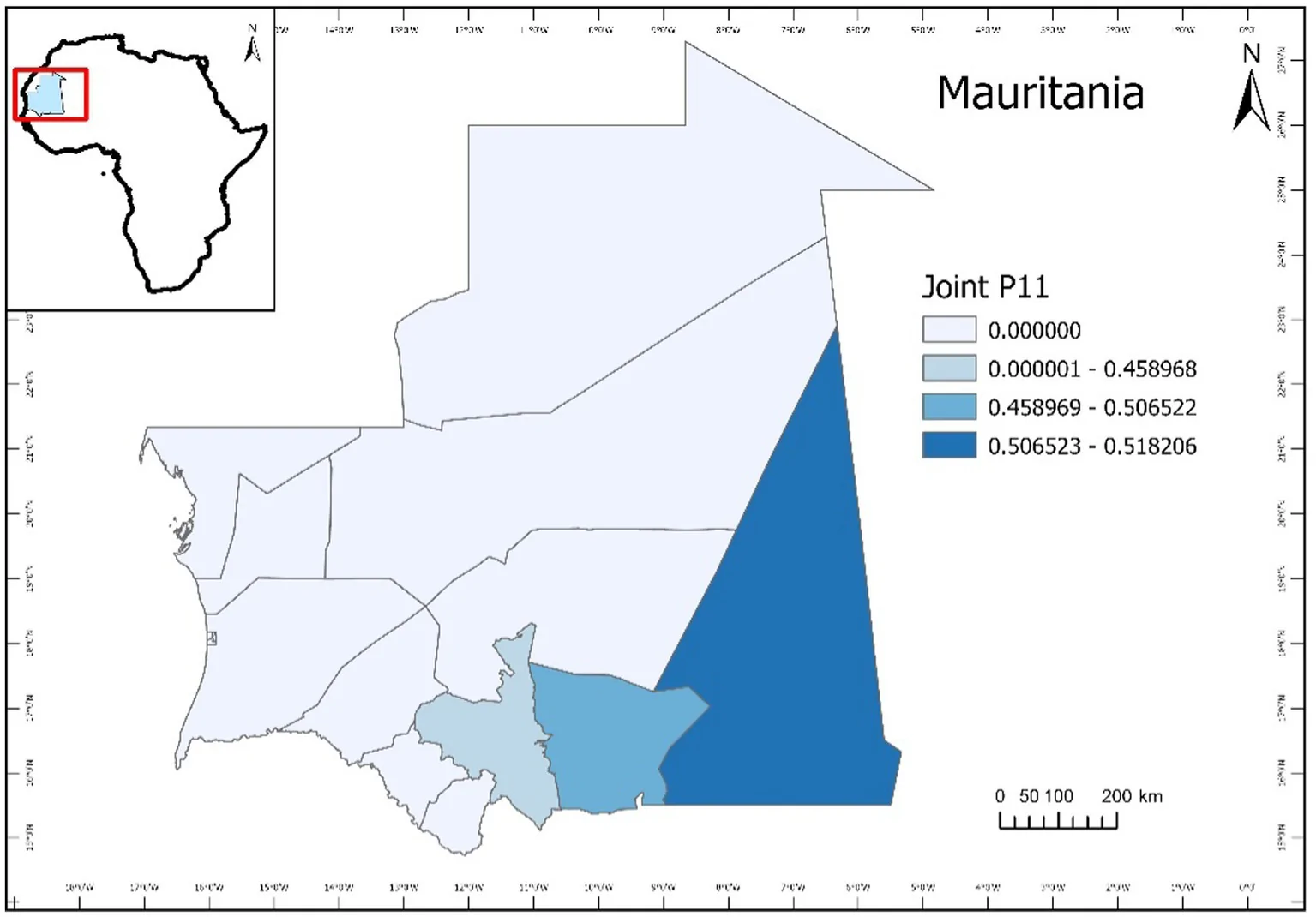
Mauritania—joint probability of a child being malnourished and anaemic.

**Figure 12:**
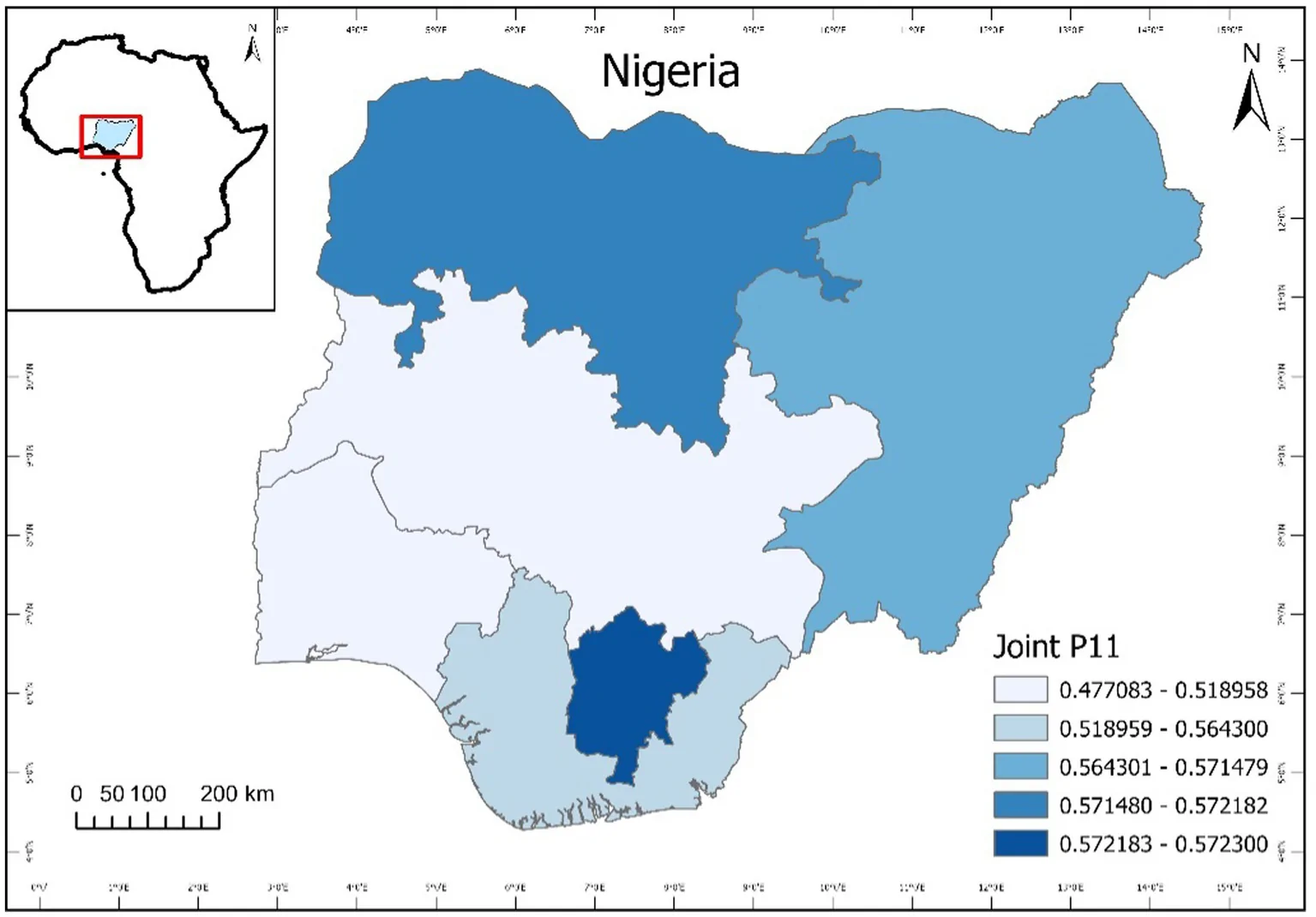
Nigeria—joint probability of a child being malnourished and anaemic.

Gambia (Figure 8), joint probabilities ranged from approximately 0.369 to 0.563. Higher probabilities were observed in central and eastern districts, while lower values were concentrated in the western coastal areas. This pattern may reflect disparities in access to nutrition and healthcare services.

Gabon (Figure 9) exhibited joint probabilities between 0.487 and 0.621. Elevated values were found in central and northern regions, suggesting localized vulnerabilities that may be linked to socioeconomic and infrastructural factors.

In Liberia (Figure 10), joint probabilities ranged from 0.469 to 0.544. Central and southeastern regions showed higher co-occurrence of malnutrition and anemia, whereas western and northern areas had relatively lower probabilities.

Mauritania (Figure 11) displayed a broader range, with some regions showing a joint probability of zero. The highest values, up to 0.518, were concentrated in central and southern areas. The presence of regions with negligible joint probabilities may indicate data limitations or genuinely low co-occurrence in those areas.

Finally, Nigeria (Figure 12) showed joint probabilities ranging from 0.477 to 0.572. Northern and central regions had higher values, while southern regions exhibited lower probabilities. These findings align with known regional disparities in nutritional outcomes and healthcare access.

Overall, the spatial heterogeneity observed across the five countries underscores the importance of region-specific public health strategies. The bivariate copula model proved effective in capturing the joint distribution of malnourishment and anemia, offering a nuanced understanding of how these two conditions co-occur geographically. By estimating joint probabilities rather than marginal risks alone, this approach allows for a more comprehensive assessment of child health vulnerabilities, particularly in regions where the burden of both conditions is compounded (20).

The maps reveal that certain regions consistently exhibit higher joint probabilities, suggesting the presence of structural or systemic factors—such as limited access to healthcare, poor dietary diversity, or socioeconomic deprivation—that may simultaneously influence both nutritional and haematological outcomes (33). These insights are critical for policymakers and health practitioners aiming to design integrated interventions that address multiple dimensions of child health. Furthermore, the identification of regions with low or negligible joint probabilities may help prioritize resource allocation and guide future data collection efforts to validate or refine these findings (3).

In summary, the use of a bivariate copula framework enhances the spatial epidemiological analysis of child health outcomes and provides actionable evidence to support targeted, data-driven public health planning.

## Discussion

This study provides important insights into the determinants of malnutrition and anemia among children in Western African countries, utilizing a bivariate copula-based approach to capture both linear and non-linear associations. Several significant predictors were identified, offering critical direction for targeted interventions.

For malnutrition, child age, gender, child weight, geographic location, and feeding practices emerged as strong determinants. Children aged 12–60 months exhibited significantly higher odds of malnutrition compared to infants under 12 months, a pattern consistent with previous findings that highlight increased nutritional vulnerability as complementary feeding practices become essential (34). The smooth term for child age confirmed a highly significant non-linear association, with risk peaking in early childhood and tapering off thereafter.

Male children were also more likely to be malnourished, aligning with regional studies suggesting biological and social vulnerabilities (35). Child weight showed a strong non-linear association with malnutrition, reinforcing the need for close monitoring even at moderate deviations from healthy weight trajectories. The smooth term for mother’s current age was also significant suggesting that younger maternal age may be associated with increased malnutrition risk.

The significant geographic variations point toward spatial clustering of malnutrition, suggesting that targeted regional programs, such as community-based nutrition rehabilitation centers, are warranted (36).

In terms of household factors, children in households using piped or well water sources, rather than bottled or sachet water, faced higher risks of malnutrition, indicating persistent water quality issues. Safe water access initiatives, such as expanded community-managed water systems and point-of-use water treatment interventions, could mitigate these risks (44). Maternal education also played a key protective role, with primary education associated with reduced malnutrition risk, reinforcing the importance of investments in female education policies. Wealth status, notably being among the richest quintile, was protective against malnutrition, underscoring the broader impact of poverty alleviation strategies on child nutrition (33). The positive associations observed for piped, natural-source, tanker, and well water relative to bottled/sachet water were counter to conventional expectations and should be interpreted cautiously. Bottled/sachet-water users may represent a relatively small and socioeconomically selected subgroup, potentially concentrated among wealthier or urban households. Although wealth index and residence type were included in the adjusted model, residual confounding and overlap among socioeconomic position, residence, country, and water-source access may remain. Moreover, the recorded primary water source does not necessarily capture reliability of supply, household storage and treatment practices, water quantity, or microbial quality at the point of consumption. The findings should therefore be understood as conditional associations with reported water-source category rather than evidence that piped, tanker, well, or natural-source water causes malnutrition.

For anemia, spatial variability, child age, child weight, and maternal age exhibited significant non-linear effects. The spatial term was highly significant, underscoring substantial geographic disparities in anemia prevalence. The smooth term for child age and mother’s current age revealed complex, non-linear relationships, suggesting that both biological and caregiving factors influence anemia risk. Urban residence was associated with a reduced risk of anemia, suggesting urban advantages in health infrastructure and food access. Children whose mothers had no formal education or only primary education were more likely to be anaemic, again demonstrating the critical role of maternal empowerment. Household economic status also mattered; children from the richest households had significantly lower odds of anemia. Dietary practices were influential: children who consumed fortified foods and eggs had notably lower odds of anemia, emphasizing the need for food fortification programs and promotion of affordable animal-source foods (37).

Given these findings, several policy recommendations emerge. First, expanding community-based nutrition programs, particularly targeting rural and impoverished areas, is essential. Programs should include regular growth monitoring, nutrition education, and promotion of nutrient-dense complementary feeding. Second, ensuring universal access to safe drinking water remains critical to reduce both malnutrition and anemia risks. Investments in rural water infrastructure and hygiene education campaigns should be prioritized (44). Third, targeted social protection interventions, such as cash transfer programs conditional on health service utilization, could help alleviate economic barriers to adequate nutrition (33). Fourth, educational policies that promote girls’ secondary school completion can have profound intergenerational impacts on child health. Finally, scaling up national food fortification programs with iron and other micronutrients, alongside community campaigns to promote the consumption of eggs and other affordable animal-source foods, should be integral to anemia prevention strategies (37).

Overall, this study highlights that addressing malnutrition and anemia in Western Africa requires a multi-sectoral approach, combining health, education, water, agriculture, and social protection policies to effectively reduce childhood undernutrition and its long-term consequences.

## Conclusion

This study examined the determinants of malnutrition and anemia among young children in selected Western African countries using a bivariate copula-based approach, providing critical insights into both individual and contextual factors influencing child health outcomes. The findings underscore the significant role of socioeconomic status, maternal education, household water sources, dietary practices, and spatial variability in shaping nutritional and hematologic outcomes. Notably, child age, weight, and gender, as well as geographic location, emerged as strong predictors of malnutrition, while maternal education, urban residence, wealth status, and specific dietary factors such as fortified food and egg consumption significantly influenced anemia risk.

The results highlight the need for integrated, multisectoral strategies to address the persistent burden of undernutrition and anemia. Policies aimed at improving access to safe drinking water, promoting maternal education, enhancing dietary diversity through fortified and nutrient-rich foods, and reducing poverty are likely to yield substantial health gains. Moreover, the strong spatial effects observed suggest that geographically targeted interventions are necessary to ensure resources reach the most vulnerable communities.

Future research should expand on these findings by exploring additional determinants of malnutrition and anemia, including more nuanced wealth indicators, family health history, and the severity of co-existing conditions such as anemia, tuberculosis, and HIV. Furthermore, factors related to preventive healthcare—such as vaccination coverage, vitamin A supplementation, deworming practices, and adherence to chronic medication regimens—warrant closer investigation for their potential influence on child health outcomes. Attention should also be given to the social aspects of malnutrition, particularly the roles of primary caregivers, healthcare chaperones, and the eligibility criteria for government economic support programs. To better capture complex and potentially nonlinear interactions among these variables, future studies could employ alternative methodologies such as structural equation modeling (SEM) or machine learning techniques like random forests and gradient boosting. Finally, longitudinal research designs are essential to track long-term trends and causal pathways in malnutrition and anemia, thereby overcoming the limitations of cross-sectional analyses and supporting the development of sustainable, evidence-driven interventions in Western Africa. The DHS sampling weights were not incorporated into the joint copula model. The reported estimates therefore describe associations within the analytical sample and may not be fully representative of the national population. Omission of the weights may affect estimated associations, predicted probabilities, and their uncertainty; consequently, the findings should be generalized cautiously. Future analyses should evaluate the robustness of the results using survey-weighted joint modeling methods. The pooled model did not include an explicit country fixed or random effect. Therefore, unmeasured differences in baseline risk across countries and survey years may have influenced the estimated associations. Furthermore, DHS wealth quintiles are survey-specific measures of relative socioeconomic rank and do not represent equivalent absolute living standards across countries. The pooled wealth coefficient should consequently be interpreted cautiously as an average association with relative national wealth position rather than as the effect of an equivalent level of material wealth across all study settings. A further limitation is that approximately 30 fixed effects and four smooth terms were evaluated for each outcome without formal adjustment for multiple comparisons. This may have increased the likelihood of chance findings, particularly among associations with *p*-values close to 0.05. The results should therefore be interpreted cautiously, with emphasis placed on effect estimates and 95% confidence intervals rather than binary significance thresholds, and marginal findings should be considered exploratory.

In conclusion, combating malnutrition and anemia in Western Africa requires a holistic approach that addresses both immediate and underlying determinants. Strengthening health systems, promoting food security, expanding educational opportunities for women and girls, and improving living conditions are essential for achieving sustained improvements in child health and development in the region.

## Data Availability

The data analyzed in this study is subject to the following licenses/restrictions: the dataset analyzed during this study is not publicly available. Additionally, further information about the data and conditions for access are available upon registration and request to DHS program-http://dhsprogram.com. Requests to access these datasets should be directed to http://dhsprogram.com.
